# Spatio-temporal modelling of under-five mortality in Lesotho using demographic and health survey data

**DOI:** 10.4314/ahs.v23i3.21

**Published:** 2023-09

**Authors:** Mthobisi Mxolisi Zondi, Henry G Mwambi, Sileshi Fanta Melesse

**Affiliations:** University of KwaZulu-Natal-School of Mathematics, Statistics and Computer Science, Pietermaritzburg, KwaZulu-Natal, South Africa

**Keywords:** Spatio-temporal modelling, under-five mortality, Integrated Laplace nested approximation, Lesotho demographic and health survey data

## Abstract

**Background:**

Lesotho is in the Sustainable Development Goal (SDG) region which aims to reduce the under-five mortality (U5M) to the average of 25 deaths per 1000 live births by the end of 2030 under the sustainable development goals (SDGs) initiative by the United Nations

**Methodology:**

This paper makes use of the Lesotho Demographic and Health Survey (LDHS dataset, which focuses on female reproductive ages 15-49 and male reproductive ages 15-54 The spatio-temporal models were used in this study to investigate how the proposed covariates change over time.

**Results:**

The results showed that children who were breastfed had a lower odd of death compared to children who were not breastfed, children from more educated mothers had significantly lower odds of U5M compared to those from less educated mothers. Having a larger number of children under the age of five also contributed significantly to an increased risk of U5M. The likelihood of U5M increased with age.

**Conclusion:**

The study recommends that mothers of under-five children be educated about breastfeeding and encouraged to use contraception in order to postpone birth and reduce parity. Rural development should be prioritized through improved primary health care; and public health services should be made more accessible to rural residents.

## Introduction

We define under-five mortality (U5M) as the probability of a child dying between birth and the age of five, expressed per 1000 live births 23. The primary goal of development agencies and international public health organizations is to reduce child mortality and improve child health 18,27. Child mortality is an indicator that can also be used to assess a population's development, health, and socioeconomic status 17. Preterm birth complications, birth asphyxia/trauma, pneumonia, congenital anomalies, diarrhoea, and malaria are the leading causes of death in children under the age of five, all of which can be prevented or treated with access to simple, low-cost interventions such as immunization, adequate nutrition, safe water and food, and quality care from a trained health provider when needed 29.

The Sustainable Development Goal (SDG) is to reduce the U5M to 25 deaths per 1000 live births. In 2019, approximately 122 countries have achieved U5M rates below the SDG target; and a further 20 countries are expected to meet the target by 2030 if current trends continue^29^. However, accelerated progress will be required in 53 countries that will not meet the target by 2030 if current trends continue. Thirty of these countries will need to double their current rate of reduction, and 23 will need to triple their current rate of reduction. Meeting the SDG target would result in an 11 million reductions in under-five mortality between 2019 and 2030. Sub-Saharan Africa and South-East Asia still require concentrated efforts to prevent 80% of these deaths.

Lesotho's under-five mortality rate is a major public health concern; Lesotho is part of the SDG region, which aims to reduce mortality 12,30. This goal appears to be unattainable because under-five mortality has fluctuated in recent years, with 113 deaths per 1000 live births in 2004, an increase of 117 deaths per 1000 live births in 2009, and 86 deaths per 1000 live births in 2014 13-15. Lesotho child mortality rate was at level of 89.5 deaths per 1000 live births in 2020, down from 90.9 deaths per 1000 live births previous year; this is a change of 1.54% 8. These findings raised concerns about whether the goal of reducing child mortality by two-thirds will be met. This question was answered in 2015, at the end of the Millennium Development Goals era. Lesotho failed to meet Millennium Development Goal 4 of the Millennium Development Goals. According to UNICEF 24, children from poor families, rural areas, and those born to mothers with no basic education have a high risk of dying before the age of five.

According to WHO 28, the Lesotho public health sector has an intervention to reduce under-five mortality: Attendance by skilled health workers during delivery increased from 61.5% in 2009 to 77.9% in 2014, while 74% of all pregnant women receive at least four antenatal care visits, and contraceptive prevalence increased to 60%. Only 68% of children under the age of one are fully immunized, falling far short of the national target of at least 80%. Individual antigens, on the other hand, show a fairly good achievement: measles 90.1%, DPT3 96%, BCG 98%, and OPV3 75.5%. Lesotho's 5-year National Strategic Development Plan 21 tries to address the challenges militating against efforts to improve child health and development. Alongside the National Strategic Development Plan, the UN has been implementing the United Nations Development Assistance Framework 22, which targets human capital strengthening through investments in health and education. The National Health Policy and the National Health Strategic Plan 23 were also developed to guide the implementation of the country's health priorities. The government is yet to approve the National Health Policy and the National Health Strategic Plan. A district readiness assessment exercise was completed to support the government's efforts to decentralize health services, and the results are being used to develop manuals, guidelines, and capacity building programs for teams at the national, district, and community levels 28.

In this paper, we discuss and fit appropriate models for performing spatio-temporal analysis of mortality among children under the age of five in Lesotho using DHS data from 2004, 2009 and 2014. These models allow us to understand how responses change over time and may provide information with significant epidemiological implications^2^. Spatio-temporal models are part of the larger class of Structured Additive Regression (STAR) models proposed by MacNab et al. 11 To handle STAR models, we used a Bayesian approach in which all unknown functions and parameters are handled as a unified general framework by assigning an appropriate prior distribution within the same general structure but with different forms and degrees of smoothness 7. Spatio-temporal models are simple extensions of basic spatial models in which a linear or non-parametric trend in time, time-space, time-covariate, and time-space-covariate interactions is included.

### Analytical Techniques

We discuss and fit appropriate models for performing spatio-temporal modelling of mortality among children under the age of five in Lesotho. The Bayesian logistic model for spatio- temporal analysis was fitted in Lesotho DHS data for 2004, 2009, and 2014. We then extend this method to allow the covariates to vary in both space and time; we use the conditional autoregressive model for space variation and the autoregressive model of order 1 for time variation. The Bayesian logistic model spatio-temporal analysis is fitted to the data using estimation techniques based on the Integrated Laplace Approximation (INLA) methods 31. These models for under-five mortality in Lesotho are then explored, with the results and outcomes discussed.

## Methods

### Data Description

The Demographic and Health Survey (DHS) is the world's largest survey, providing reliable data on child health status, health service indicators, and child and infant mortality, among other things. The dataset for this study was derived from the Lesotho Demographic and Health Survey (LDHS) data collected in 2004, 2009, and 2014. The survey was carried out in collaboration with the Lesotho Bureau of Statistics and the Inner-City Fund (ICF) Macro, which provided technical assistance. These LDHS used a two-staged sample design and were intended to allow estimates of key indicators at the national, urban, and rural levels, as well as in each of Lesotho's districts. In the first stage, 400 clusters consisting of enumeration areas were chosen from 2006 population Census areas for 2009 and 2014. (94 and 118 in the urban; 306 and 284 in the rural respectively). In contrast, with 405 clusters, 2004 followed the 1996 population Census frame (109 in the urban and 296 in the rural areas). The second stage included a systematic household sampling. A three-model questionnaire for DHS was considered, which included men, women, and households.

The LDHS 2014 focused on female reproductive ages (15-49) and men ages 15-54. In this study, we used the child file, which was obtained from a total sample of 6621 mothers who were interviewed. Only 3,138 children were chosen from the individual female sample. Because the primary focus of this study is child mortality, the focus was only on women who responded to child mortality questions. Information on children who died was compiled using data from all births to women in the five years preceding the survey. Cases were chosen from the entire sample to include only women who reported having given birth in the five years preceding the survey (3138 participants). In the child file, responses were ordered in reverse chronological order, with the most recent birth appearing first. As a result, the models fitted to the data were based on 3138 children. The 2004 LDHS included 7095 interviews with women of reproductive age. In this study, we chose 3598 survey respondents who answered the child question. In comparison, we selected 3880 participants from the 2009 LDHS out of a total sample of 7,624.

### Study variables

#### Outcome variable

The outcome variable in this study is child survival status of a child which is dichotomous variable showing the status: of a child alive or not. The response variable is coded as “1” if the child is dead and 0 otherwise the time of the survey.

#### Explanatory variables

This study's data set included both categorical and continuous variables. The variables were divided into two categories: demographic and social characteristics. The procedure we followed to select explanatory variables is documented elsewhere 31. In summary, it was discovered that the level of education, place of residence, current breastfeeding status, and the number of children aged 5 and under were all related to child survival status. Age was also found to have a non-linear effect on child survival status, so it's continuous form (mean = 4.43 and standard deviation = 6.833 for 2004; mean = 5.27 and standard deviation = 8.215 for 2009; and mean = 4.88 and standard deviation = 7.663 for 2014) was used in subsequent analyses.

### Statistical model

Let *yijt* be the child survival status for child *j* at time *t* in district i:i = 1,2,…,10 such that *y_ijt*=0 if child j in district i is alive at time t 1 and otherwise. This study assumes *y_ijt* the dependent variable is univariate Bernoulli distributed, i.e. *y_ijt \p_ijt~Bernoulli*(*p_ijt*). The unknown mean response E(*y_ijt)=p_ijt* may relate to the independent variables as follows:



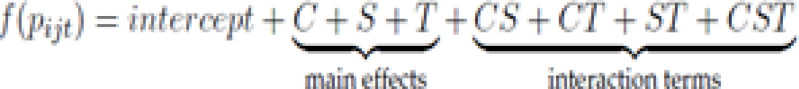



Where the function f(.) is a logit link function in the context of a GLM, the intercept term gives the initial amount of risk shared by all individuals, districts and time. The main effects C; S and T designates the covariate, spatial and temporal effects, respectively. The second-order interaction terms CS; CT; ST describes the contribution to the risk due to a mixture of main effects that cannot be resolved additively by main effect, CST represents the covariate-space-time interaction 10,20.

### The effect of covariates

Covariates were introduced in this paper to improve the small area predictions. Thus, the coefficients should be interpreted with caution, as they depict environmental associations and are not indicative of causal pathways or individual-level risk determinants 10. Broadly, covariates significantly associated with under-five mortality included: demographic characteristics and social factors.

### Parameter Estimation

In this study, we used the fully Bayesian estimation procedure where parameters were assigned prior distributions as will be discussed in the prior's specification section. Final parameter estimates were obtained from the posterior distribution.

### Prior Parameter Distribution

Based on the information of various research sources, more suitable user-defined hyper priors have been given using appropriate expressions in INLA. Specifically, the non-informative normal distribution prior was used for the fixed effects while a random walk model of order 2 was used for the continuous covariate, namely age. The temporal effects were modelled by a first-order autoregressive process allowing for correlation between the two periods and the districts. The spatial components were the CAR model for the structured random effects.

### Posterior Distribution

The posterior distribution is obtained after combining the prior distribution with the observed data. In this paper, we used the fully Bayesian strategy and to obtain inferences for the latent Gaussian models was performed through the R software using INLA package.

### Application/Data analysis

In this paper, we apply two models, specifically; a spatial model (Model 1) and a spatiotemporal model (Model 2). The spatio-temporal model captures covariate space-time interaction. These are,

Model 1


$$logit(p_{ijt})=\beta_{0}+f(age)+Z_{ijt}^{T}\gamma +v_{i}$$

Model 2


$$logit(p_{ijt})=\beta_{0}+f(age)+Z_{ijt}^{T}\gamma_{t}+v_{i}$$

where,

-β_0, is the intercept describing the logit under-five mortality rate when all covariates have a zero value

-f(age), represents a function of age. Here age was taken as a continuous and we assume it has a non-linear effect on child survival status

-$Z_{ijt}^{T}$, represents the vector of the categorical covariates for child *j* living in province *i* at time *t*, with *y* representing the regression coefficients for the spatial model while *y t* represents the time-dependent regression coefficients modelled using the autoregressive model of order 1 given by: : *X_i_ = αX_i_*_−1_ + *ε_t_* where *ε_t_* is a zero-mean white noise

-v_i, represents the structured random effects in both models.

Model 1 This is the simplest model which does not allow for any interaction i.e. covariate-time, space-time, covariate-space or space-covariate-time. It is a model of continuous covariate age modelled with a random walk model of order 2 as explained by Lindgren, F et al. 5 which is assumed to have a non-linear effect on U5M, categorical covariates which are considered to have a linear effect on child status and the structured random effects modelled using the conditional autoregressive model (CAR).

Model 2 This is the more complex model that provides for space-covariate-time interaction and all two-order interactions. U5M is modelled as a non-linear the function of age using the random walk model of order 2, while the rest of the covariates are modelled as functions of time and space using the autoregressive processes of order 1 and the conditional autoregressive model respectively. The model incorporates the structured random effects which cater for any unobserved covariates which vary spatially across the districts. There are numerous formulations of the spatio-temporal models that do exist with no conventional one. It is also a challenge or not easy to justify any single formulation of the spatio-temporal models on empirical grounds. Our study applies and compares Models 1 and 2, and warn that these models are not in themselves conclusive.

## Results

The categorical covariates were assumed to have a linear effect on the logit of probability of U5M. Three proposed covariates were found to have no association with the probability of U5M in the year 2004, besides the type of residence which showed that urban children had higher odds of U5M than rural area children (OR=1.57, 95%, 1.06, 2.08) these ambiguous results might be due to that LDHS 2004 dataset had a quality issue 15. In the periods 2009 and 2014 U5M for children living in urban areas was significantly lower than those residing in rural areas (OR=0.44, 95%, 0.29, 0.60) and (OR=0.41, 95%, 0.22, 0.59) respectively. The study by Fink, G. et al. 6 verifies that an increase in urbanization lessens U5M because of the high standard of living. Hence the study by Motsoene^16^ stated that urbanization in Lesotho has been doubling every seven years. In the period 2009 and 2014, Children who were currently breastfed were less likely to contribute to U5M than those who were not being breastfed (OR=0.50, 95%, 0.32, 0.68) and (OR=0.43, 95%, 0.22, 0.64), respectively. The lower odds in 2014 could depict an increase in breastfeeding overtime. The results showed that in the year 2009 and 2014 children of mothers with secondary and higher education have significantly low odds of U5M than those with none and primary education (OR=0.52, 95%, 0.34, 0.69) and (OR=0.43, 95%, 0.30, 0.68), respectively. Additionally, the data also told us that in the period 2009 and 2014, the more the children aged below 5 in the household, the higher the risk of U5M (OR=0.34, 95%, 0.17, 0.52) and (OR=0.41, 95%, 0.18, 0.62), respectively. Our findings suggest a time-dependent covariate effect where the benefits of urbanization, current breastfeeding, education and number of children aged 5 and below is model pronounced when in later surveys to earlier ones. The findings seem to agree to the study by Li et al. 9, which gave decreasing U5M from 1990 to 2015.

### The effect of age

**Figure 1:**
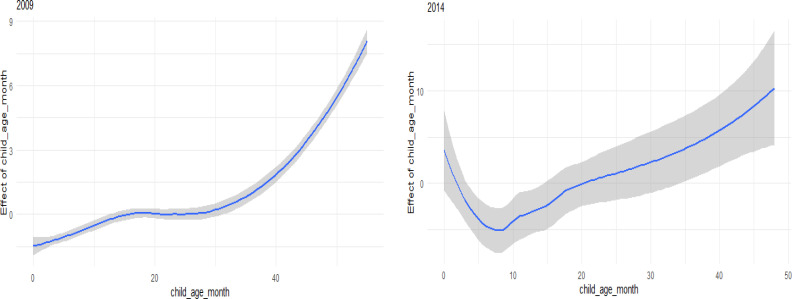
Non-linear effect of age at death of the two time periods (based on Model 1)

Figure 1 shows that the likelihood of U5M increased with age at death. The effect is linear from birth to 30 months and increasing after 30 months toward 60 months, and this is displayed when using 2009 data. When using 2014 data, the effect decreases from birth until 12 months and increasing impact after 12 months toward 60 months. This increasing of U5M with rising age could be as a result of nutrient scarcity in their diet which may render them vulnerable to many infectious and other diseases than those below 12 months who were still likely to be breastfed entirely and under maternal protection.

### Spatio-temporal effect

We review in brief the spatio-temporal evolution of Model 2 concerning some several risk factors, particularly the place of residence, current breastfeeding, level of education and number of children aged 5 or under.

### Under-five Mortality in Lesotho

Since the covariates in 2004 period have non-significant effect when fitting model 1, therefore we will proceed analysing the data using 2009 and 2014 Lesotho Demographics and Health survey data set.

**Figure 2:**
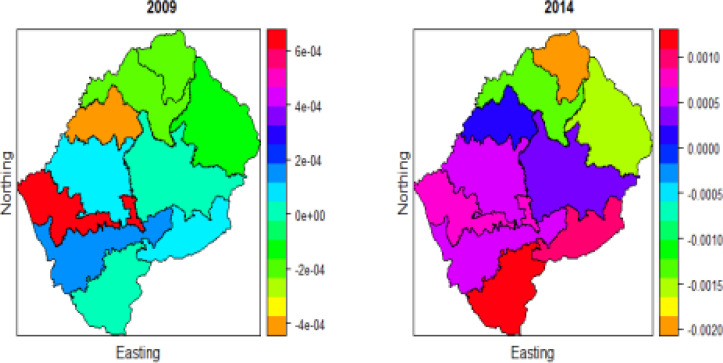
Spatio-temporal distribution of under-five mortality in Lesotho for two periods 2009 and 2014

Figure 2 displays the Spatio-temporal distribution of U5M in years 2009 and 2014 surveys. The year 2009 has low risk in Berea (shown in orange colour) and highest in Mafikeng (shown in red colour). In both these years, the risk is little in the North, North-East, and North-West of Lesotho. Butha-Buthe (shown in orange colour) has the lowest risk in the year 2014 and Maseru has moderate (shown in navy colour). Overall, there was a massive change in the spatial pattern of U5M in the study period. Previous studies 5, 9, 19 indicated that the spatial variation of U5M risk fluctuates significantly overtime.

### Place of residence

**Figure 3:**
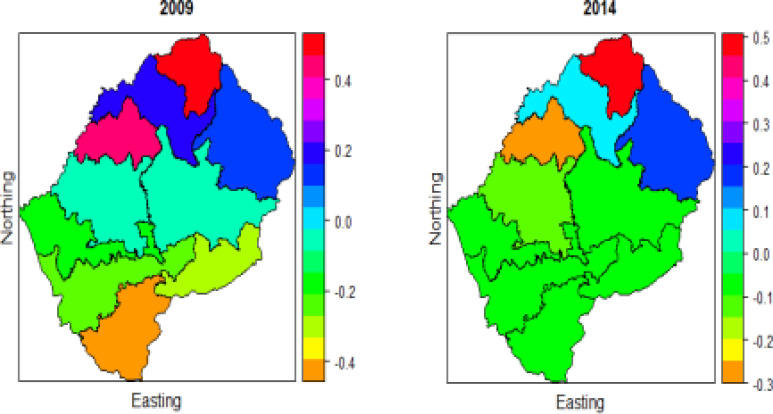
Spatio-temporal effects of place of residence on under-five mortality for the two time periods 2009 and 2014

Figure 3 displays the effects of place of residence on U5M for the two-time period. The spatial pattern for these two periods appears to have some difference, the effect of residence in the central, South, South-East, South-West, East and West (shown by orange, green and light blue) is low and high in the South and South-West (shown in red, pink and navy). Butha-Buthe, Quthing and Berea appear to experience the highest effect of residence, and this might be due to urbanization since these districts are less urbanized, as discussed earlier in Table 1.

**Table 1:** The estimated OR with the corresponding 95% credible intervals for the spatial (based on Model 1)

Covariates	Year 2004	2009	2014
**Residence** (Rural)	1	1	1
Urban	1.57(1.06;2.08)	0.44(0.29;0.60)	0.41(0.22;0.59)
**Current Breastfeeding** (No)	1	1	1
Yes	1.09(0.6;1.58)	0.50(0.32;0.68)	0.43(0.22;0.64)
**Education** (Non & Primary)	1	1	1
Secondary & Higher	1.08(0.70;1.72)	0.52(0.34;0.69)	0.50(0.30;0.68)
**No. Of Children Below 5**(2 or more)	1	1	1
Less than 2 children	1.11(0.69;1.72)	0.34(0.17;0.52)	0.41(1.18;062)

### Current breastfeeding

**Figure 4:**
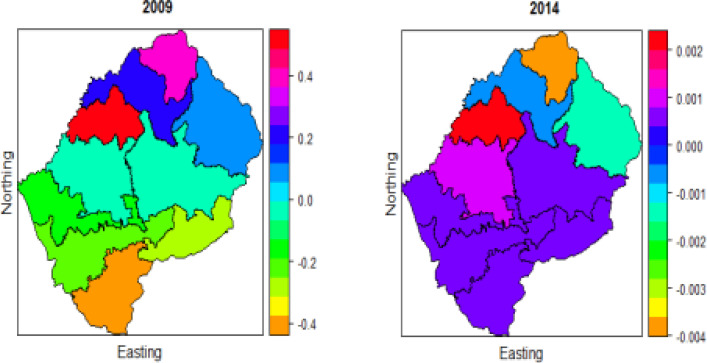
Spatio-temporal effects of Current breastfeeding on under-five mortality for the two periods 2009 and 2014

Figure 4 shows that the current breastfeeding effect on U5M is high in the North-East and with time. increases over time. The choropleth maps show that current breastfeeding has a greater effect on U5M in 2014 than in 2009. The World Health Organization and the United Nations Children's Fund Infant and Youth have trained a large number of health workers and counselors, and the course has been adapted to respond to specific needs in Lesotho by training those who care for mothers and children in the basics of good infant and young child feeding, which is why more mothers in Lesotho are breastfeeding^22^. It was low from the center to the south, south-east, southwest, west, and east in 2009. The breastfeeding effects on U5M was low from the center to the south, south-east, south-west, west and east in 2009. The effect was minimal in Butha-Buthe (shown in orange) in 2014, and minimal in Quthing in 2009. Berea appears to have the greatest effect for both study periods (shown in red). Generally, children from Berea have a lower deprivation rate than children from other districts; they also have longer breast-feeding duration than children from other districts 23-24. The study by Mokoena 12 also confirms that there has been a decrease in U5M due to breastfeeding in in many Sub-Saharan regions since 1999.

### Education

**Figure 5:**
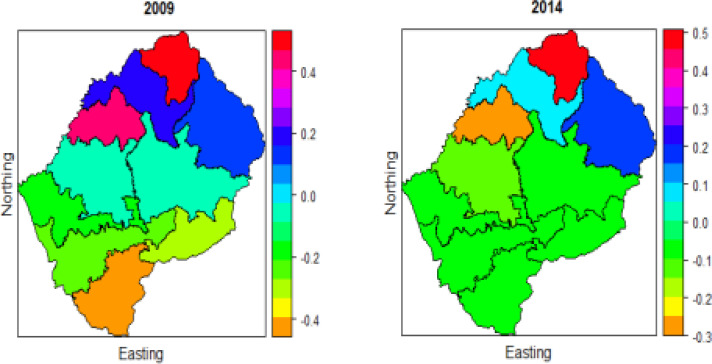
Spatio-temporal effects of education on under-five mortality for the two periods 2009 and 2014

The choropleth in Figure 5 shows that the spatial pattern depicted by education changes significantly over time; this also agrees with the study by De Onis et al. 4 and Badji 1, which revealed that education has a significant negative effect on U5M over time. Generally, the highest effect of education is in the North, North-West, and the West of the country in the years 2009 and 2014. A large proportion of the population lives on Lesotho's western border, which is where the majority of economic activity occurs. As a result, people in this part of Lesotho are more educated than the rest of the country, and new development begins here before spreading to other parts of the country 3. All of the initiatives to combat under-five mortality can also be found in this region of Lesotho.

### Number of children 5 or under

**Figure 6:**
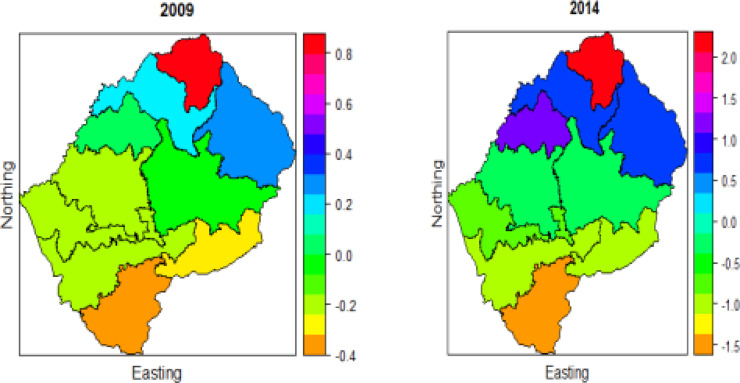
Spatio-temporal effects of Number of children 5 or under on under five-mortality for two periods 2009 and 2014

Figure 6 displays that the spatial pattern of 2009 and 2014 data have small differences, in both periods, Butha-Buthe has the highest effect, Quthing experience the smallest effect number of children 5 or under on U5M. Central through South- East, South-West, East, and West has the smallest effect; and North, North-East, and North-West have a high effect of children 5 or under on U5M. As explored earlier in Table 1, that number of children aged below 5 has a significant effect on U5M over time, and the choropleth maps also give similar results for the three periods.

### Under-five Mortality in Lesotho when considering Survey complexity

**Figure 7:**
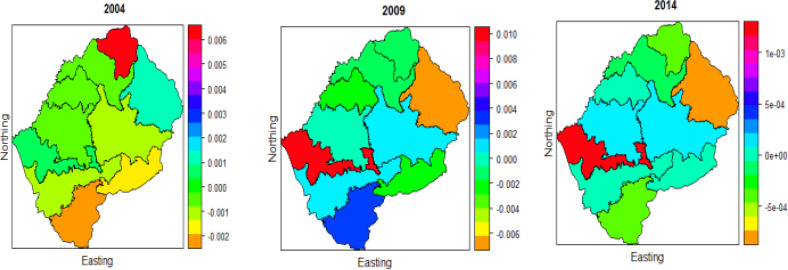
The risk of under-five mortality for the three-period 2004, 2009 and 2014

**Figure 8:**
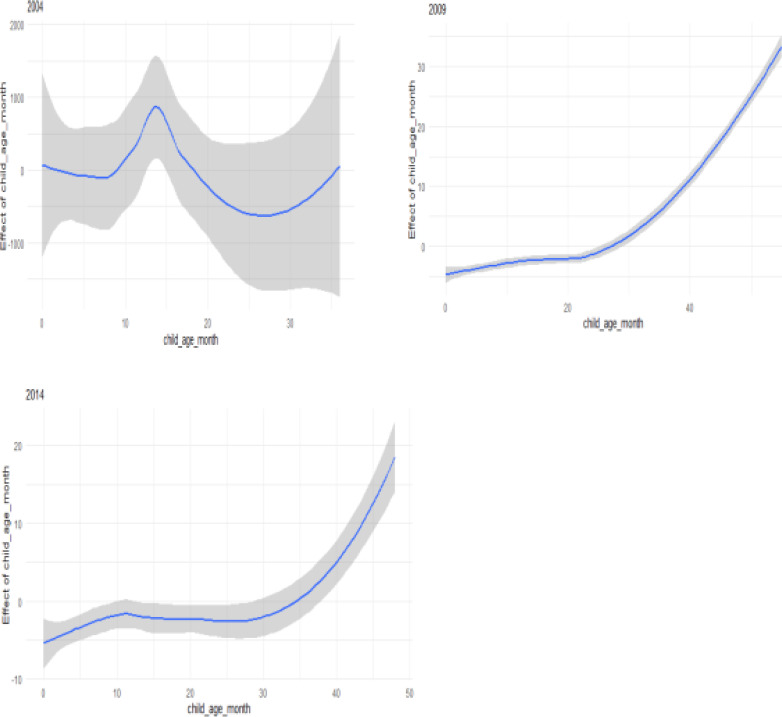
Spline showing effect of age at death on under-five mortality for the years 2004, 2009 and 2014 respectively

Table 2 shows that the effect of all covariates is statistically significant in the three periods 2004, 2009, and 2014. The results indicate that the children who were breastfed had lower odds of death in the three periods compared to those who were not breastfed (OR=0.43, 95%, 0.18, 0.67), (OR=0.36, 95%, 0.17, 0.56) and (OR=0.37, 95%, 0.14, 0.60), respectively. Being more educated also showed a significant protective effect in the three periods on U5M (OR=0.21, 95%, 0.026, 0.438), (OR=0.25, 95%, 0.05, 0.46) and (OR=0.33. 95%, 0.11, 0.55), respectively. Having many children aged below 5 contribute to the higher odds of U5M (OR=0.40, 95%, 0.17, 0.63), (OR=0.34, 95% 0.15, 0.54) and (OR=0.34, 95%, 0.10, 0.59), respectively for three periods. The children from urban areas had better odds of surviving than those from rural areas (OR=0.44, 95%, 0.19, 0.69), (OR=0.46, 0.95%, 0.23, 0.70) and (OR=0.50, 95%, 0.34, 0.83), respectively for three periods. The choropleth maps in Figure 7 reveals that the year 2004 has a completely different spatial pattern compared to 2009 and 2014, which appears to have a minimal difference in their spatial pattern. In general, we can see significant changes of U5M risk pattern overtime. The results further display the disparity within districts of Lesotho, this agrees with the study by Li, Z et al. 9 who used Bayesian hierarchical models in modelling U5M rate in Kenya using the 1980-2014 DHS data, showed that there is a sharp decline in U5M rate, but large variability in subnational level. Berea (shown in red colour) is shown as having a high effect of U5M, and the lowest effect is observed in Quthing (displayed in orange colour) for the year 2004. Mokhotlong (displayed in orange colour) appeared to experience a low effect of U5M in the 2009 and 2014 periods; Mafikeng (displayed in red colour) has a high effect. In the year 2004, the lowest effect is observed in the central, through Western, Eastern and Northern parts of Lesotho. There is a moderate effect in the Southern part in 2009. The year 2004 had a low effect in the central to Western and Southern parts of Lesotho, and the highest effect is perceived in Northern, and the moderate effect is observed in Eastern and West-East. The first plot in Figure 8 above, the plots correspond to the period of 2004 that shows that age at death has a negative effect on child mortality, there is a peak at 13 months and increasing effect 36 months toward 50 months. The plot for 2009, which is the middle in Figure 8 reveals that the effect is linear from birth until 25 months and increasing from 25 months toward 50 months. The plot that corresponds to 2014 shows that the effect is linear from birth until 35 months and rising from 35 months toward 50 months.

**Table 2:** The estimated OR with the corresponding 95% credible intervals for the spatial (Model 2)

Covariates	Year 2004	2009	2014
**Residence** (Rural)	1	1	1
Urban	0.44(0.19;0.69)	0.46(0.23;0.70)	0.50(0.34;0.83)
**Current Breastfeeding** (No)	1	1	1
Yes	0.43(0.18;0.67)	0.36(0.17;0.56)	0.37(0.14;0.60)
**Education** (Non & Primary)	1	1	1
Secondary & Higher	0.21(0.026;0.44)	0.25(0.05;0.46)	0.33(0.11;0.55)
**No. Of Children Below 5**(2 or more)	1	1	1
Less than 2 children	0.40(0.17;0.63)	0.34(0.15;0.54)	0.34(0.10;0.59)

## Discussion

In this paper, we presented a full Bayesian analysis to carry out spatial and Spatio-temporal modelling of under-five mortality (U5M) in Lesotho using 2004, 2009, and 2014 LDHS data. We assumed that all the covariates had a linear effect on U5M except age which was assumed to have a non-linear relationship and was modelled using a random walk of order 2 model 4. The results showed that the proposed covariates in 2004 were insignificantly associated with U5M, so only in 2009 and 2014 were significantly associated with U5M using a model that ignored survey complexity. When we considered interaction between space-time and covariate, the results revealed that the effect of covariates was significantly associated with U5M for the three study periods. The study, however, establishes that age had a positive linear relationship with U5M, as shown in Figure 1 and 8. A possible reason for the linearly increasing effect could be that children aged below the age of one are more likely to be still breastfed and therefore have stronger immune systems. With increasing age, they are more likely to be weaned as breastfeeding is minimized or terminated altogether, and this lessens their immunity, thus making them more vulnerable to many childhood diseases. Also, a study by the United Nations Children's Emergency Fund (UNICEF) reported a high U5M of stunted children in Lesotho. This was compounded by the reliance of mothers from rural areas on traditional healers or untrained nursing staff to take care of their children, which could aggravate the health of the children's health 10. Children living in urban areas were less likely to die at the age below five than those living in rural areas. We can suggest that individuals living in urban areas are frequently better economically than their rural counterparts. The children who are breastfed showed an improving odds ratio in the three periods over those who are not breastfed. Education also showed that it has a significant effect over time on U5M. Having many children aged below 5 contributes to U5M. Spatial effects in the model account for unobserved effects and also help to model effects that vary spatially. Identifying high U5M areas may help in informing intervention strategies for those districts. Figure 7 showed the spatio-temporal distribution of U5M in the three periods. These patterns are essential in that they show how U5M is changing for different periods and may help reveal new patterns and risk factors. The maps show a pattern of reduction of U5M overtime. The spatio-temporal model in this study extends the existing Spatio-temporal models by combining both temporally and spatially varying coefficients. This helps in observing not only how the response variable changes over time but also the effects of the coefficients on the response variable change over time.

## Conclusion

This study investigated the existing statistical models for mapping and modeling as used in the Bayesian framework and adopted the recently advanced INLA package that is found in R software. We then applied these models to under-five mortality (U5M). The proposed models only catered for areal (lattice) data, Geostatistical, and point pattern data were not considered in this paper. The data used in this study were taken from Lesotho Demographic and Health Surveillance for 2004, 2009, and 2014. The non-linear effects of age at death of an under-five child were modelled using the random walk of order 2 (RW2), while the spatially structured effects and spatially unstructured effects in the model were modelled using the Gaussian Markov Random Field and a zero-mean Gaussian process respectively. The study revealed that the spatio-temporal model demonstrated a significantly low effect of the proposed cavariates overtime. The study recommends that mothers of under-five children be educated about breastfeeding and encouraged to use contraception in order to postpone birth and reduce parity; rural development should be prioritized through improved primary health care; and public health services should be made more accessible to rural residents. Additionally, a positive linear effect due to age at death to U5M was shown, which might be due to children increasingly being weaned from breastfeeding as they grow. In the future, we can take into consideration the full multivariate and the spatio-temporal case, which has become an exciting research avenue. With increasing computing power such models which require efficient computing power can now be handled. In the future, we can also make HIV adjustment since Lesotho has a relatively high prevalence of HIV, to avoid serious bias in estimates of U5M particularly before antiretroviral therapy (ART) treatment became widely available. Additionally, we can also estimate the trend in the U5M for the Lesotho area using the DHS data set.
